# Chromosome structural variation of two cultivated tetraploid cottons and their ancestral diploid species based on a new high-density genetic map

**DOI:** 10.1038/s41598-017-08006-w

**Published:** 2017-08-09

**Authors:** Wen-wen Wang, Zhao-yun Tan, Ya-qiong Xu, Ai-ai Zhu, Yan Li, Jiang Yao, Rui Tian, Xiao-mei Fang, Xue-ying Liu, You-ming Tian, Zhong-hua Teng, Jian Zhang, Da-jun Liu, De-xin Liu, Hai-hong Shang, Fang Liu, Zheng-sheng Zhang

**Affiliations:** 1 Engineering Research Center of South Upland Agriculture, Ministry of Education, Southwest University, Chongqing, 400716 China; 2 State Key Laboratory of Cotton Biology/Cotton Research Institute, Chinese Academy of Agricultural Sciences, Anyang, 455000 China

## Abstract

A high-resolution genetic map is a useful tool for assaying genomic structural variation and clarifying the evolution of polyploid cotton. A total of 36956 SSRs, including 11289 released in previous studies and 25567 which were newly developed based on the genome sequences of *G. arboreum* and *G. raimondii*, were utilized to construct a new genetic map. The new high-density genetic map includes 6009 loci and spanned 3863.97 cM with an average distance of 0.64 cM between consecutive markers. Four inversions (one between Chr08 and Chr24, one between Chr09 and Chr23 and two between Chr10 and Chr20) were identified by homology analysis. Comparative genomic analysis between genetic map and two diploid cottons showed that structural variations between the A genome and At subgenome are more extensive than between D genome and Dt subgenome. A total of 17 inversions, seven simple translocations and two reciprocal translocations were identified between genetic map and *G. raimondii*. Good colinearity was revealed between the corresponding chromosomes of tetraploid *G*. *hirsutum* and *G*. *barbadense* genomes, but a total of 16 inversions were detected between them. These results will accelerate the process of evolution analysis of *Gossipium* genus.

## Introduction

Cotton (*Gossypium* spp.) is one of the world’s most important crops, with a wide variety of applications in the textile and paper industries, home fixtures, medical supplies, chemicals and oil^1^. The *Gossypium* genus is currently comprised of 52 species, including 45 diploids classified into 8 genome designations (A through G, and K) and 7 allotetraploids designated AD^2, 3^. Allotetraploid *Gossypium* are classic natural allotetraploids that derived from trans-oceanic dispersal of an A-genome ancestral African species resembling *G. herbaceum* (A_1_) or *G. arboreum* (A_2_), and its hybridization with a native American D-genome species resembling *G. raimondii*, followed by divergence from their common ancestor^4^. Although the two cultivated allotetraploids, *G. barbadense* and *G. hirsutum*, may share a common progenitor, the two species differ substantially in many ways.

In the past few years, several cotton genome sequences have been released, including diploids *G. arboreum* (A_2_) and *G. raimondii* (D_5_), and allotetraploids *G. hirsutum* (AD_1_) and *G. barbadense* (AD_2_)^5–11^. A series of structural variations have been detected among these genomes based on comparative analyses. *G. raimondii* and *G. arboreum* chromosomes 1, 4–6, and 9–13 were highly colinear, whereas large-scale rearrangements were observed on chromosomes 2 and 3 of *G. raimondii*, and deletions/insertions were observed on chromosomes 7 and 8 of *G. arboreum* ^7^. Subsequently, 9 translocations and 28 inversions were identified by comparing the genome sequence of *G. hirsutum* ^8^ with *G. raimondii* ^5^. In addition, with the release of the *G. barbadense* genome sequence^11^, SNPs were identified by comparing the diploid progenitors (A_1_, A_2_, and D_5_) and the tetraploid *G. hirsutum* (AD_1_) with the At and Dt subgenomes of *G. barbadense* (AD_2_)^11^. The distribution of SNPs showed that the genomic divergence between the two tetraploids (*G. barbadense* and *G. hirsutum*) was less than the divergence between the tetraploid and its diploid progenitors.

A high-resolution map is another way to study the diversity of genome structure and the morphological diversity of a genus and its biology. In recent years, many interspecific genetic maps have been developed from crosses between *G. hirsutum* and *G. Barbadense* ^12–22^. Among these maps, only six possess more than 2000 markers^12, 15, 18, 19, 21, 22^.Rong *et al*.^12^ reported several inversions and two reciprocal translocations between At and A chromosomes. Guo *et al*.^13^ identified translocations between some chromosomes (Chr02 and Chr03, Chr04 and Chr05) based on homologous analysis, corroborating the result reported by Rong *et al*.^12^ Wang *et al*.^23^ reported five inversions (Chr15-D02, LGA03-D07, Chr.12-D08, Chr.04-D09, Chr.10-D11) between a whole-genome DNA marker map and the genetic map released by Rong *et al*.^12^. Shi *et al*.^21^ identified a few minor discrepancies in some marker orders along the D genome and the Dt or At subgenomes. Based on a high density genetic linkage map, Li *et al*.^22^ reported that only a portion of Chr10 and Chr20 showed colinearity, whereas Chr07 and Chr16 had good homology but little colinearity. In order to support assembly of a precise genome of allotetraploid cotton, a sequence-based interspecific genetic map composed of 4,999,048 SNP loci was constructed, revealing some structural variations in the At genome and Dt genome, including 15 simple translocations reported for the first time^20^.

It has become evident that higher-order genomic architectural features can lead to susceptibility to DNA rearrangements^24, 25^. Genomic rearrangements can cause Mendelian diseases or complex traits such as behaviors, or they can represent benign polymorphic changes. Revealing variation of genome architecture between the two tetraploid cultivated cottons will further deepen our understanding of the molecular basis of fiber formation. The release of genome sequences together with the high-density map is valuable for understanding genome structure and for exploring the genetic basis of important agronomic characters.

In this study, a new high-density genetic linkage map for tetraploid cotton was constructed based on SSR markers. This study aimed to (i) design SSR markers based on the sequence of two diploid species *G. arboreum* ^7^ and *G. raimondii* ^6^; (ii) construct a new high-density genetic map; (iii) explain the relationship between genetic map and four sequenced species of cotton by aligning sequences of polymorphic loci to the genome of cotton; and (iv) reveal genome structural differences by comparing physical maps of two tetraploid cottons.

## Results

### Marker information

In the present study, a total of 22567 new primers were designed according to the reference genomes of *G. raimondii* ^6^ (SWU10001-SWU22560) and *G. arboreum* ^7^ (CCRI00001-CCRI13007) (Supplementary Table 1–3). The 13007 primers from *G. arboreum* ^7^ were distributed over all 13 chromosomes, and the highest marker density (16.74/Mb) was on Chr13. For 12560 markers from *G. raimondii* ^6^, the number of SSR markers on the chromosome ranged from 852 (Chr13) to 1323 (Chr09). Chr12 (27.35/Mb) and Chr03 (25.22/Mb) had the higher marker densities. Dinucleotide and pentanucleotide repeats were the most common SSR type in both *G. arboreum* ^7^ and *G. raimondii* ^6^, followed by trinucleotide repeats (Supplementary Figure 1).

A total of 36956 markers, including 22567 newly developed primers and 11389 markers from cottongen (http://www.cottongen.org), were screened for polymorphic primers between CCRI35 and Pima S-7. Different series of primers showed different polymorphism ratios (Supplementary Table 4). For example, among 3926 NAU markers, 918 (23.4%) showed polymorphism, and generated 1028 loci, whereas 302 (9.5%) of 3177 HAU SSRs were polymorphic. For the newly developed markers in this study, 12560 SWU and 13007 CCRI SSRs generated 1432 and 1762 polymorphic SSRs with a polymorphism ratio of 11.40% and 13.60%, respectively. In total, 5394 primers showed clear polymorphism, for a polymorphism ratio of 14.60%, and generated 6022 polymorphic loci.

### Construction of a genetic map

Based on the 6022 polymorphic loci, a genetic map with 6009 loci was constructed. The total length of this map was 3863.97 cM with an average of 0.64 cM between adjacent markers. The At genome contained 3049 loci with 1932.50 cM in total length and an average of 0.63 cM between adjacent markers, whereas the Dt genome contained 2960 loci with 1931.47 cM in total length and an average of 0.65 cM between adjacent markers. The chromosomes with the most loci were Chr05 (337 loci) and Chr19 (329 loci), whereas Chr04 (147 loci) had the fewest, with an average of 231 loci on each chromosome. The longest chromosome was Chr05 (236.42 cM), and the shortest was Chr04 (96.77 cM), with an average length of 148.62 cM (Supplementary Figure 2, Supplementary Table 5). In addition, among 6022 polymorphic loci, 307 (5.11%) loci showed segregation distortion (P < 0.05), including 104 on the At genome and 203 on the Dt genome (Supplementary Table 5). The largest number of distorted loci were on Chr02 (70), accounting for 34.48% of all distorted loci.

Regarding the 603 primers that segregate at multiple loci in the genetic map, most pairs of loci were mapped on homologous chromosomes (Supplementary Figure 3). All homologous chromosomes showed high colinearity. However, four inversions were identified in the present study: One inversion each was detected between homologous chromosomes Chr08 and Chr24, and Chr09 and Chr23, respectively. Two inversions were identified between Chr10 and Chr20. In addition, the two large well-known reciprocal translocations between Chr02 and Chr03 and between Chr04 and Chr05 were also identified.

### Colinearity between the genetic map and the physical maps of two tetraploid cultivated cottons

To study the colinearity and genome variations, the physical maps of 26 chromosomes were constructed by blasting the polymorphic loci of the genetic map against the reference genome sequence of *G. hirsutum* ^8^ and *G. barbadense* ^10^. Among the 6009 loci distributed on 26 chromosomes, 5442 (90.56%) and 5121 (85.22%) were anchored on the genome of *G. hirsutum* ^8^ and *G. barbadense* ^10^, respectively (Supplementary Figure 3). The average physical distance between adjacent markers is 0.32 Mb and 0.34 Mb, giving an overall density of 3 loci/Mb and 3 loci/Mb for *G. hirsutum* ^8^ and *G. barbadense* ^10^, respectively (Supplementary Table 5).

The relationship between the genetic map and two tetraploid genomes was explored on the basis of this genetic map and two physical maps of *G. hirsutum* ^8^ and *G. barbadense* ^10^. All chromosomes showed high colinearity, except for a segment of Chr06 showing poor colinearity with corresponding chromosomes in *G. barbadense* ^11^ (Supplementary Figure 3). However, two inversions were identified between Chr05 (172.52–173.60 cM) and Gh_A05 (59.98–72.30 Mb), Gb_A05 (58.73–66.37 Mb); and between Chr08 (63.17–64.25 cM) and Gh_A08 (53.18–86.89 Mb), Gb_A08 (50.65–84.82 Mb) after comparing the genetic map and two physical maps of tetraploid cottons. In addition, 4884 of 6009 loci anchored on both *G. hirsutum* ^8^ and *G. barbadense* ^10^ were used for analyzing the genomic colinearity of two cultivated tetraploid cottons. All chromosomes of *G. hirsutum* showed good colinearity with corresponding chromosomes of *G. barbadense* except for a segment of A06. Furthermore, at least 16 inversions were identified that differentiated the *G. hirsutum* and *G. barbadense* genomes (Supplementary Table 6).

### Comparison of the genetic map and two diploid genomes

Sequences of 3868 and 4205 markers were homologous between the physical maps of *G. arboreum* ^7^ and *G. raimondii* ^6^, with alignment proportions of 64.37% and 69.97%, respectively. We found a high degree of colinearity between the genetic map and the *G. raimondii* ^6^ genome (Supplementary Figure 4), but poor colinearity between the genetic map and the *G. arboreum* ^7^ (Supplementary Figure 5). Therefore, only the *G. raimondii* ^6^ genome was used for further comparative genomic analysis. We identified 2 reciprocal translocations, 17 inversions and 7 simple translocations between this genetic map and the D genome after comparative analysis (Supplementary Table 7).

We were surprised to find that a segment of Chr02 and a segment of Chr03 of genetic map were anchored on an overlapping region (17.16 Mb − 31.31 Mb) of Gr_Chr05 (Supplementary Table 8). Therefore, the sequence of this overlapping region from *G. raimondii* ^6^ was used to anchor to *G. hirsutum* ^8^. Further analysis showed that a total of 8 fragments (4 fragments from A02 and 4 from A03) are cross-distributed in this overlapping region of Gr_Chr05 (Supplementary Table 9).

## Discussion

Due to their high level of polymorphism and codominant inheritance, SSR (Simple Sequence Repeat) markers are a useful tool for a variety of applications including study of genetic variation, gene mapping, construction of linkage maps, and marker assisted selection. Although a number of SSRs for cotton have been developed from small segments of genomic libraries or EST sequences^26–28^, these generally provided uneven and incomplete genome coverage. The release of cotton genome sequences allows us to mine SSRs at the genomic level, and analyze their distributions, putative functions, and evolution. Totals of 13007 and 12560 SSRs were designed on the basis of reference sequences of *G. arboreum* ^8^ and *G. raimondii* ^6^, and the markers collectively covered approximately 99.92% and 99.77% of the physical length of the genomes, respectively. Among the 25567 SSR markers, 3138 showed polymorphism between CCRI35 and Pima S-7. Therefore, the newly developed SSR markers in the present study were applicable for map construction, fine mapping, map-based cloning and molecular marker assisted breeding. In addition, compared with publicly available markers (http://www.cottongen.org), the primers designed in the present study were endowed with precise information about their chromosomal affiliation and physical position. This new information provides us a convenient approach to unravel the relationship between diploid and tetraploid genomes or the homology between genetic and physical maps.

It is well known that species resembling *G. herbaceum or G. arboreum* and *G. raimondii*, respectively, were the putative contributors of the At and Dt subgenomes of tetraploid cotton^29^. These diploid genomes share a high level of synteny or colinearity with the corresponding subgenomes in tetraploid cotton^8–11^. In this study, comparative genomic analysis showed that colinearity between D and Dt genomes is much higher than that between A and At genomes. Possible reasons are that much more structural change exists between A and At genomes than between D and Dt genomes^8^, or that much more misassemble exists in the genome sequence of *G. arboreum* ^7^ than *G. raimondii* ^6^.

Some rearrangements between the genetic map and *G. raimondii* ^5^ identified in this study were slightly different from previous reports^20^. Two new inversions between Chr05 and Gr_Chr09 and between Chr08 and Gr_Chr04 were identified in this study. Moreover, the inversions were also found when we compared this genetic map with two physical maps of tetraploid cottons, which may be caused by the difference between parents and germplasm used for sequencing or a result of misassembles in the reference genome sequence (Supplementary Figure 3, Supplementary Table 7). Moreover, in addition to the rearrangements between Chr22 and Gr_Chr09, rearrangement between Chr04 and the same position of Gr_Chr09 was also identified between genetic map and *G. raimondii* ^6^ (Supplementary Table 7). Rearrangements between Chr02/Chr17 and Gr_Chr03, between Chr09/Chr23 and Gr_Chr06, between Chr11/Chr21 and Gr_Chr03 and between Chr13/Chr18 and Gr_Chr01 were also identified in the present study. Several researches considered the phenomenon as simple translocation. However, the existence of rearrangement in both At subgenome and Dt subgenome reveal that it may be due to misassembles of the *G. raimondii* ^6^ genome or the difference between *G. raimondii* ^6^ and its ancestor, rather than simple translocation between *G. raimondii* and the tetraploid genome reported by Zhang *et al*.^8^ and Wang *et al*.^20^.

Although *G. barbadense* ^10^ and *G. hirsutum* ^8^ are thought to share a common progenitor, intense directional selection by humans and the environment has led to different yield and/or quality characteristics between two tetraploid cottons. The release of genome sequences makes it easy to study genomic divergence between *G. barbadense* ^10^ and *G. hirsutum* ^8^. However, little literature reports the difference in genome structure between two cultivated tetraploid cottons^10, 11^. In the present study, comparative genomic analysis showed that the 26 chromosomes of *G. hirsutum* showed good colinearity with corresponding *G. barbadense* chromosomes. However, some rearrangements were still detected between the corresponding chromosomes of the two tetraploid cottons. In the *G. barbadense* ^10^ genome, at least 16 large inversions (>1 Mb) were identified, compared with the genome of *G. hirsutum* ^8^ (Supplementary Table 6). It predicted that these structural variations occurred after polyploidization, perhaps in association with directional selection by humans and/or the environment. An important subject for further investigation is whether these structural variations are linked to agronomic and/or quality traits that distinguish the two cultivated cotton species. Exploring these structural variations may facilitate understanding of genome evolution and gene/QTL cloning from the corresponding orthologous regions.

Two large reciprocal translocations between Chr02 and Chr03 and between Chr04 and Chr05 were identified when comparing either *G. arboreum* ^7^ or *G. raimondii* ^6^ with genetic map, consistent with the result of Brubaker *et al*.^30^. Furthermore, Chr02, Chr03, Chr04 and Chr05 showed good colinearity between two tetraploid cottons. Collectively, these findings indicated that chromosomal translocations between Chr02 and Chr03 and between Chr04 and Chr05 may occur after polyploidization and before divergence of the two tetraploid cottons.

It is worth noting that an overlapping region (16.77 Mb-31.31 Mb) on Gr_Chr05 was found after comparing Chr02/Chr03 with Gr_Chr05 and the segments of Chr02 and Chr03 anchored on Gr_Chr05 are cross-distributed in this overlapping region. We hypothesize that the reason for this phenomenon is the difference between Ga_Chr02 and Gr_Chr05 or chromosome rearrangement of the Ga_Chr02 before allopolyploidization. In other words, the fragments for A02 or A03 of tetraploid got together across multiple rearrangements (inversion or simple translocation) in this overlapping region before allopolyploidization. Then, this region was divided into two segments which were assembled on corresponding chromosomes after reciprocal translocations (Supplementary Figure 6).

Wang *et al*. proposed a new chromosome nomenclature for tetraploid cotton and established 13 homeologous chromosome pairs^31^. They treated Chr02/Chr14 and Chr03/Chr17 as homeologous chromosome pairs. After comparative analysis between genetic map and *G*. *raimondii* ^6^, we found that the proportion of segments on Chr02 corresponding to Gr_Chr03 is larger than that corresponding to Gr_Chr05, and the proportion of segment on Chr03 corresponding to Gr_Chr05 is larger than corresponding to Gr_Chr03. The same results had been reported by Wang *et al*.^20^. These findings support that the homologous chromosome of Chr02 is Chr17, whereas the homologous chromosome of Chr03 is Chr14, which had been applied in previous studies^19, 32^. Therefore, we consider that the current released chromosome names A02 and A03 should be interchanged.

## Materials and Methods

### Plant materials

*G. hirsutum* cv. CCRI35 as a recurrent parent was crossed with *G. barbadense* cv. Pima S-7 as a donor to establish a BC1 population. CCRI35, a high yielding and disease resistant cultivar, has been widely planted in China in the past decade^33^. Pima S-7 is a *G. barbadense* cultivar that is photoperiod insensitive and produces high-quality fiber in USA^34, 35^. Subsequently, the BC1 population [(CCRI35 × Pima S-7) × CCRI35] including 94 indivaduals was used as the mapping population.

### Development of SSRs

To construct a high-density genetic map, in addition to markers released by previous studies (http://www.cottongen.org), approximately 1000 primer pairs on each chromosome were designed from *G. raimondii* ^6^ and *G. arboreum* ^7^. The reference genome sequences of *G. raimondii* ^6^ were retrieved from phytozome (https://phytozome.jgi.doe.gov/Graimondii_er) and *G. arboreum* ^7^ were retrieved from cottongen (http://www.cottongen.org). Simple sequence repeat (SSR) primers of *G. arboreum* ^7^ and *G. raimondii* ^6^ were designed using SSR locator 1^36^. The microsatellite motifs were searched by the following criteria: eighteen repeat units for mononucleotide (Mono) repeats, nine for dinucleotide (Di) repeats, six for trinucleotide (Tri) repeats, four for tetranucleotide (Tetra) repeats, three for pentanucleotide (Penta) repeats and three for hexanucleotide (Hexa) repeats. The major parameters for designing SSR primers were: (1) primer length from 18 to 27 bases; (2) PCR product size ranging from 100 to 200 bp; (3) melting temperature between 55 and 65 °C with 60 °C being the optimum annealing temperature; (4) GC content of 45–65% with an optimum of 50%. SSR primers developed form *G. raimondii* were named ‘SWU’, whereas primers designed with *G. arboreum* were named ‘CCRI’. All primers were synthesized by Invitrogen Co. Ltd. (Shanghai, China).

### DNA extraction, PCR amplification, and electrophoresis

Total genomic DNA from fresh young leaves of the parents and 94 BC_1_ individuals were extracted according to a modified CTAB method^37^. All SSR primer pairs, including 11289 released by previous studies and 25567 developed in this study, were screened for polymorphism between the mapping parents and those showing clear polymorphism were used to genotype the BC_1_ population. PCR amplification and product tests were performed according to Zhang *et al*.^37^. Loci detected were named with the primer name. For multiple loci revealed by the same primer pair, an extra letter was added to the primer name, such as a/b/c, indicating the molecular size from smallest to largest.

### Map construction

The molecular map was generated using JoinMap 4.0 with the parameter that the logarithm of odds (LOD) threshold was ≥4.0^38^. Map distances in centiMorgans (cM) were calculated using the Kosambi mapping function^39^. The genetic linkage map was drawn with Mapchart 2.0^40^. In addition, each segregating marker was tested by a Chi-squared test to determine if it deviated significantly from the expected Mendelian segregation ratio.

### Colinearity and comparative genomic analysis among a genetic map and genome of cotton

The sequence of the *G. arboreum* ^7^, *G. raimondii* ^6^ *and G. barbadense* ^10^ and primers were obtained from cottongen (http://www.cottongen.org), while *G. hirsutum* ^8^ were retrieved from phytozome (https://phytozome.jgi.doe.gov/Ghirsutum_er). The sequences of polymorphic markers were carried out BLASTN to find corresponding chromosome and position on genome of *G. arboreum*, *G. raimondii*, *G. hirsutum* and *G. barbadense* ^6–8, 10^. The best hit was selected to analyze the relationship between the genetic map and physical map.

The relationship between genetic map and two diploid cottons was illustrated intuitively using the online drawing tool circos-0.67 (http://circos.ca/). In addition, markers anchored on both *G. hirsutum* ^8^ and *G. barbadense* ^10^ were used to analyze the relationship of two tetraploid cottons. The colinearity was illustrated intuitively using the online drawing tool D3 (https://github.com/d3/d3/wiki).

## Electronic supplementary material

Supplementary Information

Supplementary Table 1

Supplementary Table 2
