# Supplementary material for: Chromosome structural variation of two cultivated tetraploid cottons and their ancestral diploid species based on a new high-density genetic map
Source: Sci Rep. 2017 Aug 9;7:7640. doi: 10.1038/s41598-017-08006-w (PMC5550419; doi:10.1038/s41598-017-08006-w)
Supplement: Supplementary file 1 — Supplementary Information [file 41598_2017_8006_MOESM1_ESM.docx]

**Chromosome structural variation of two cultivated tetraploid cottons and their ancestral diploid species based on a new high-density genetic map**

Wen-wen Wang^1, *^, Zhao-yun Tan^1, *^, Ya-qiong Xu^1, *^, Ai-ai Zhu^1^, Yan Li^1^, Jiang Yao^1^, Rui Tian^1^, Xiao-mei Fang^1^, Xue-ying Liu^1^, You-ming Tian^1^, Zhong-hua Teng^1^, Jian Zhang^1^, Da-jun Liu^1^, De-xin Liu^1^, Hai-hong Shang^2^, Fang Liu^2^ , Zheng-sheng Zhang^1^

^1^Engineering Research Center of South Upland Agriculture, Ministry of Education, Southwest University, Chongqing 400716, China, and ^2^State Key Laboratory of Cotton Biology/Cotton Research Institute, Chinese Academy of Agricultural Sciences, Anyang 455000, China. ^*^These authors contributed equally to this work. Correspondence and requests for materials should be addressed to F.L. (email:liufcri@163.com) or Z.S.Z. (email: zhangzs@swu.edu.cn)


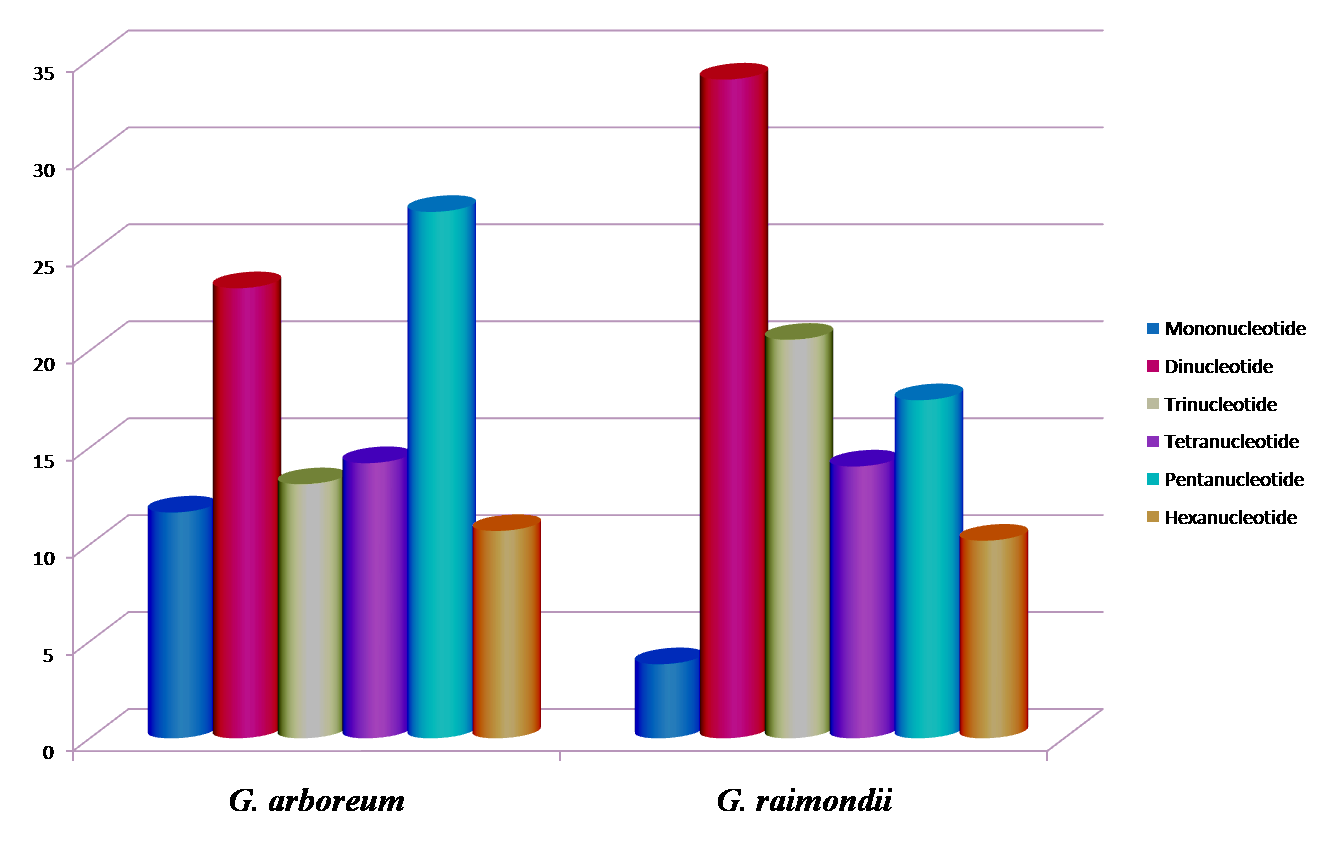


Supplementary Figure 1

**Relative frequencies (%) of SSR types developed in this study.**

Supplementary Figure 2

**Genetic map constructed in this study.**


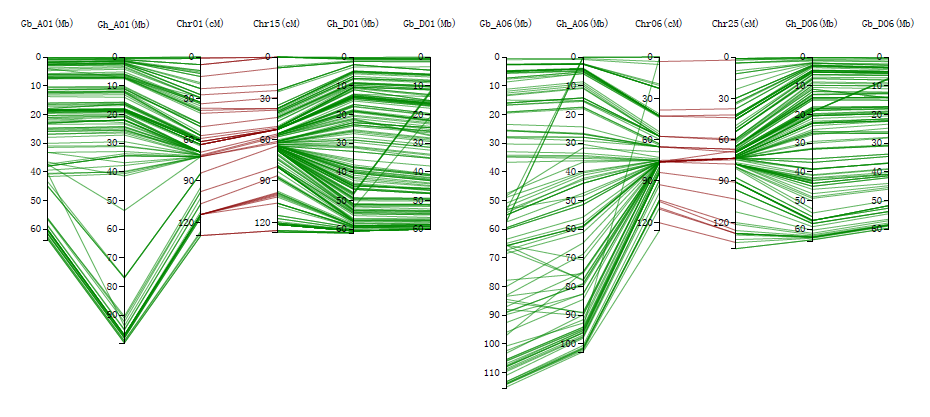

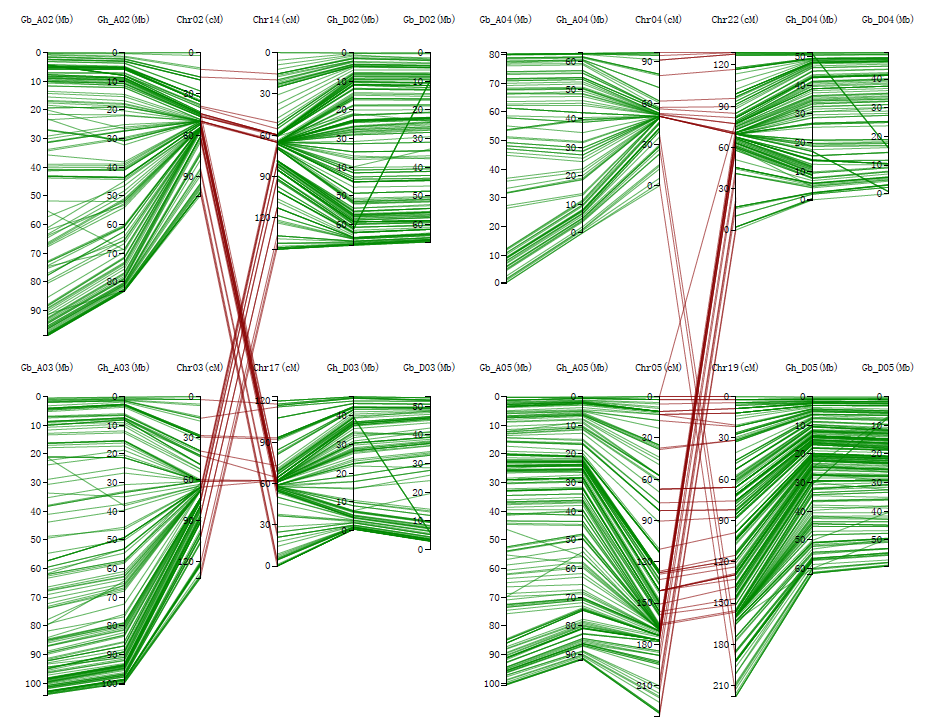

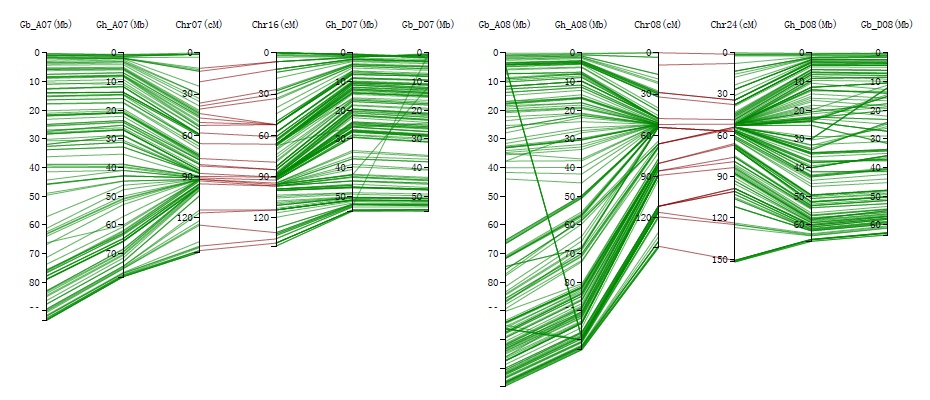


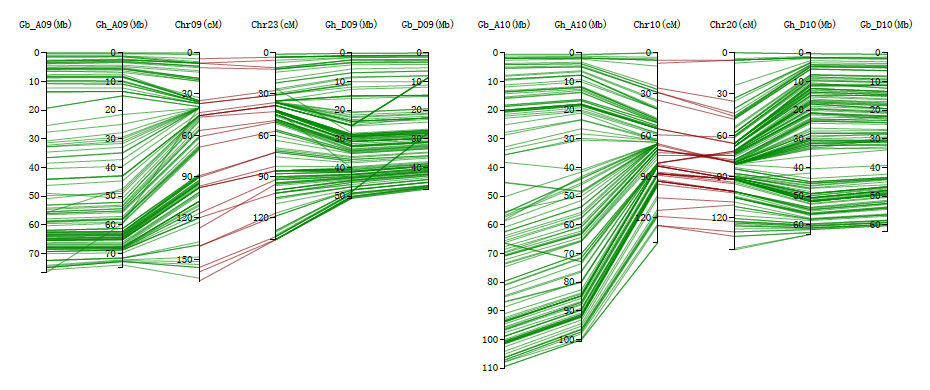

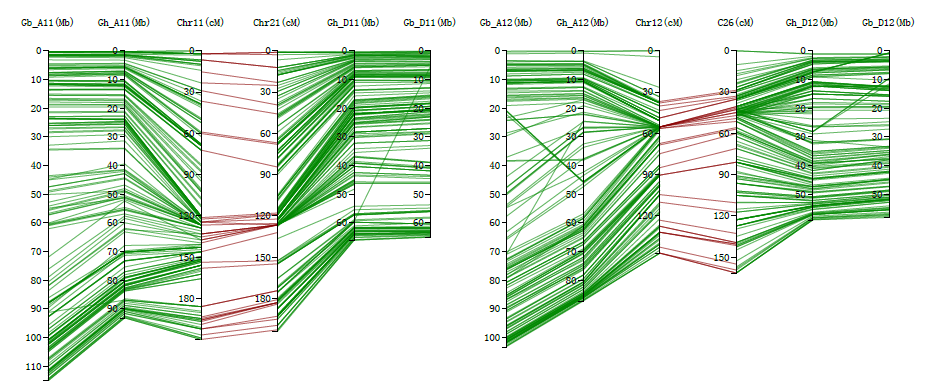

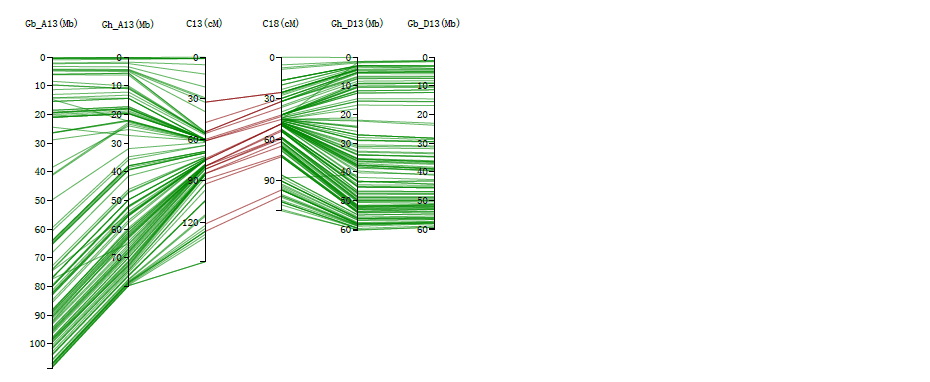


Supplementary Figure 3

**Intuitive diagram of colinearity of homologous chromosomes and the relastionship of the genetic map and physical map.**

Colinearity between homologous chromosomes of genetic map and between the genetic map and the physical maps of two tetraploid cultivated cottons.The brown lines represent collinearity of homologous chromosomes and the green lines represent collinearity between genetic map and two physcial maps.


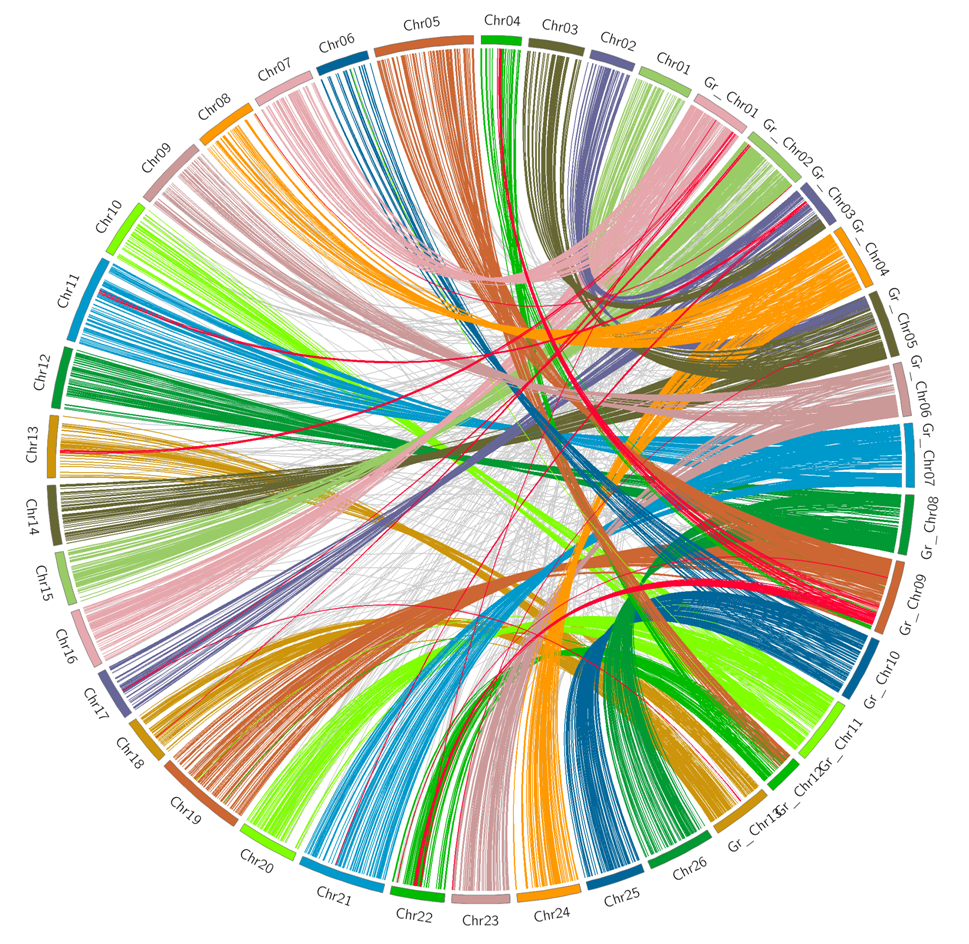


Supplementary Figure 4

**Intuitive diagram of affinity between the genetic map and *G. raimondii* genome.**

Comparative genomics analysis between genetic map and *G. raimondii* genome**.** Red lines represent simple translocations.


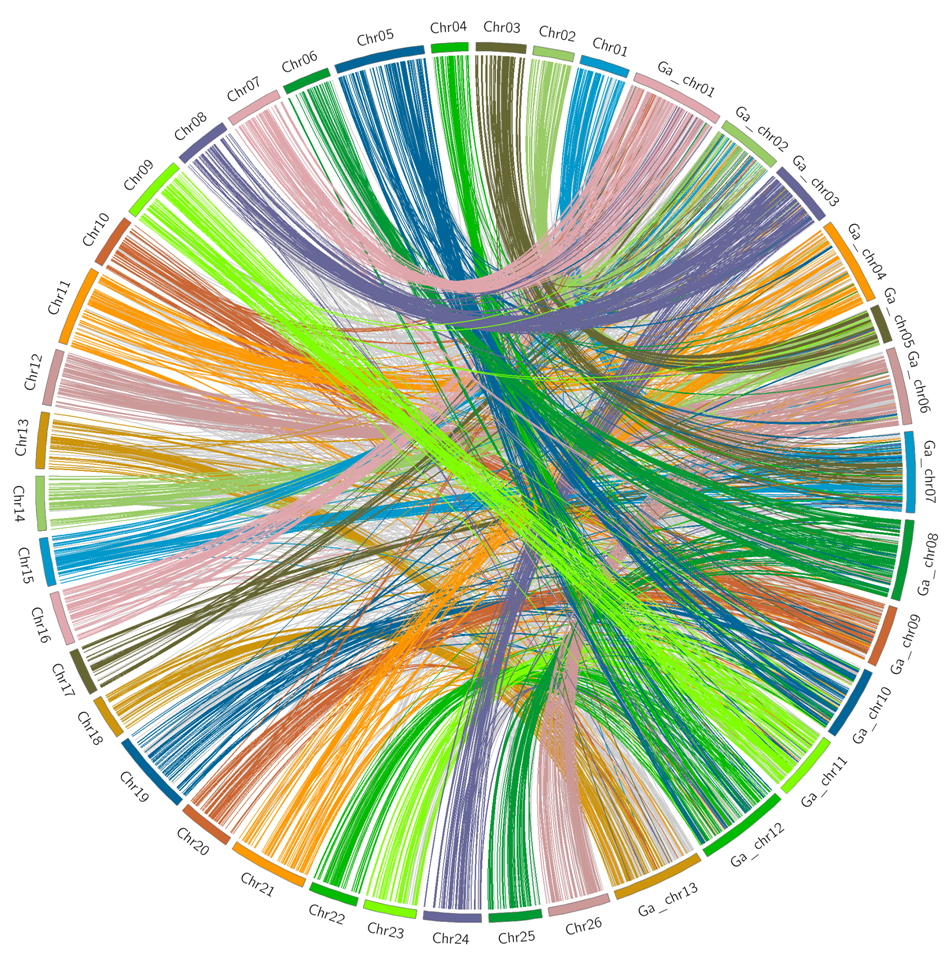


Supplementary Figure 5

**Intuitive diagram of affinity between the genetic map and *G. arboreum* genome.**

Comparative genomics analysis between genetic map and *G. arboreum* genome**.**


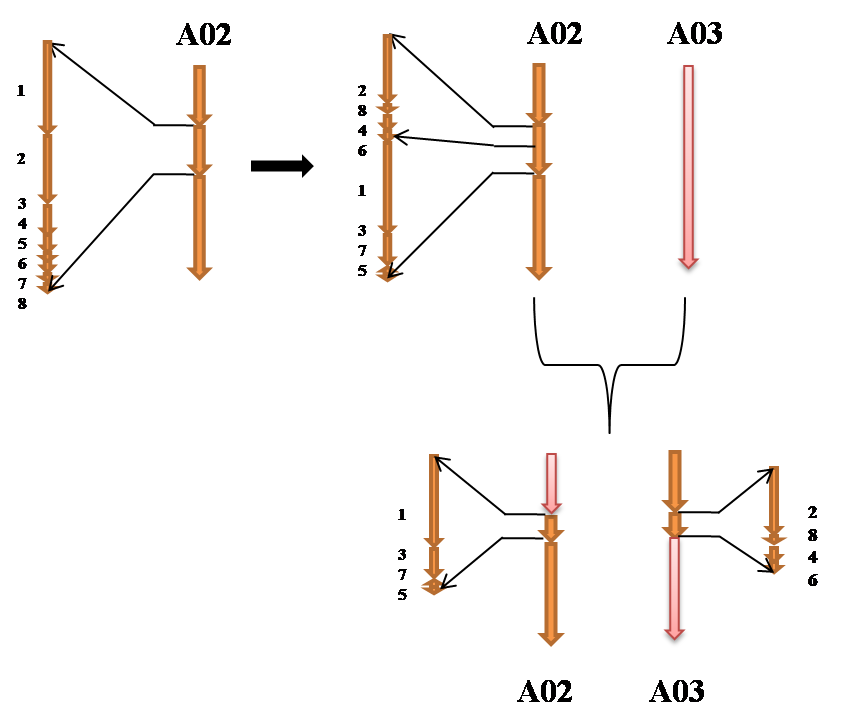


Supplementary Figure 6

**A model for the evolution of chromosomes A02 and A03.**

**Supplementary Table 1-9**

**Supplementary Table 1. Primers developed based on genome sequences of *G. raimondii* (Separate file).**

**Supplementary Table 2. Primers developed based on genome sequences of *G. arboreum*(Separate file).**

**Supplementary Table 3. Distribution of SSRs among chromosome sequences of *Gossypium arboreum* and *Gossypium raimondii.***

**Supplementary Table 4. Polymorphism radio of various primers and the number of loci produced by these polymorphic primers.**

**Supplementary Table 5. Characteristics of the genetic map and corresponding physical map of two tetraploid cultivated cottons.**

**Supplementary Table 6. Inversions between *G. hirsutum*(AD1) and *G. barbadense*(AD2) genomes.**

**Supplementary Table 7. Genome variations identified via comparative mapping against D-genome pseudomolecules.**

**Supplementary Table 8. Breaking manner of two reciprocal translocations.**

**Supplementary Table 9. Distribution of the overlapping region of A02 and A03 compared with Gr_Chr05 after allopolyploidization.**

| **Supplementary Table 1. Primers developed based on genome sequences of *G. raimondii* (Separate file).**  **Supplementary Table 2. Primers developed based on genome sequences of *G. arboretum* (Separate file).**  **Supplementary Table 3. Distribution of SSRs among chromosome sequences of *Gossypium arboreum* and *Gossypium raimondii.*** | | | | | | | | | | |
| --- | --- | --- | --- | --- | --- | --- | --- | --- | --- | --- |
|  |  |  |  |  |  |  |  |  |  |  |
|  | ***G. arboreum*** | | | |  |  | ***G. raimondii*** | | | |
| **Chr** | **SSR No.** | **Density** | **Cover** | **Coverage** |  | **Chr** | **SSR No.** | **Density** | **Cover** | **Coverage** |
|  |  | **(SSR/Mb)** | **Length(Mb)** | **(%)** |  |  |  | **(SSR/Mb)** | **Length(Mb)** | **(%)** |
| ca01 | 1000 | 6.80 | 147.01 | 99.92 |  | Chr01 | 897 | 16.06 | 55.40 | 99.16 |
| ca02 | 1000 | 6.85 | 145.97 | 99.97 |  | Chr02 | 923 | 14.70 | 62.67 | 99.84 |
| ca03 | 1000 | 7.26 | 137.52 | 99.89 |  | Chr03 | 1154 | 25.22 | 45.74 | 99.95 |
| ca04 | 1000 | 7.83 | 127.48 | 99.81 |  | Chr04 | 916 | 14.73 | 62.09 | 99.85 |
| ca05 | 1001 | 6.94 | 144.01 | 99.90 |  | Chr05 | 860 | 13.41 | 63.95 | 99.70 |
| ca06 | 1001 | 8.73 | 114.56 | 99.89 |  | Chr06 | 881 | 17.25 | 50.91 | 99.68 |
| ca07 | 1002 | 9.73 | 102.89 | 99.95 |  | Chr07 | 1022 | 16.76 | 60.92 | 99.89 |
| ca08 | 1001 | 10.36 | 96.57 | 99.97 |  | Chr08 | 982 | 17.19 | 56.98 | 99.74 |
| ca09 | 1000 | 8.24 | 121.33 | 99.99 |  | Chr09 | 1323 | 18.71 | 70.58 | 99.81 |
| ca10 | 1001 | 9.47 | 105.61 | 99.92 |  | Chr10 | 893 | 14.36 | 62.08 | 99.84 |
| ca11 | 1001 | 7.86 | 127.27 | 99.91 |  | Chr11 | 888 | 14.17 | 62.57 | 99.82 |
| ca12 | 1001 | 9.91 | 100.95 | 99.93 |  | Chr12 | 969 | 27.35 | 35.39 | 99.88 |
| ca13 | 999 | 16.74 | 59.62 | 99.89 |  | Chr13 | 852 | 14.61 | 58.21 | 99.82 |
| Total/Mean | 13007 | 8.98 | 117.75 | 99.92 |  | Total/Mean | 12560 | 17.27 | 57.50 | 99.77 |

| **Supplementary Table 4. Polymorphism radio of various primers and the number of loci produced by these polymorphic primers.** | | | | |
| --- | --- | --- | --- | --- |
|  |  |  |  |  |
| **Primer** | **Primer No.** | **Polymorphic No.** | **Rel freq(%)** | **Loci_No.** |
| BNL | 692 | 222 | 32.08 | 239 |
| CCRI | 13007 | 1762 | 13.55 | 1961 |
| CIR | 392 | 119 | 30.36 | 137 |
| CGR | 1244 | 211 | 16.96 | 232 |
| DOW | 100 | 47 | 47.00 | 50 |
| DPL | 849 | 195 | 22.97 | 229 |
| Gh | 700 | 106 | 15.14 | 120 |
| HAU | 3177 | 302 | 9.51 | 322 |
| JESPR | 309 | 80 | 25.89 | 98 |
| NAU | 3926 | 918 | 23.38 | 1028 |
| SWU | 12560 | 1432 | 11.40 | 1606 |
| Total | 36956 | 5394 | 14.60 | 6022 |
|  |  |  |  |  |

| **Supplementary Table 5. Characteristics of the genetic map and corresponding physical map of two tetraploid cultivated cottons.** | | | | | | | | | | |
| --- | --- | --- | --- | --- | --- | --- | --- | --- | --- | --- |
|  |  |  |  |  |  |  |  |  |  |  |
| **Chr.** | **Loci No.** | **Genetic Distance (cM)** | **Average distance (cM)** | **Physical Distance of Gh (Mb)** | **Average distance of Gh (Mb)** | **Maker density of Gh (No/Mb)** | **Physical Distance of Gb (Mb)** | **Average distance of Gb (Mb)** | **Maker density (No/Mb)** | **Segregation distortion** |
| Chr01 | 172 | 129.71 | 0.75 | 103.46 | 0.60 | 1.66 | 63.39 | 0.37 | 2.71 | 0 |
| Chr02 | 243 | 106.76 | 0.44 | 90.21 | 0.37 | 2.69 | 98.71 | 0.41 | 2.46 | 70 |
| Chr03 | 257 | 132.42 | 0.52 | 108.61 | 0.42 | 2.37 | 103.38 | 0.40 | 2.49 | 0 |
| Chr04 | 147 | 96.77 | 0.66 | 82.30 | 0.56 | 1.79 | 79.71 | 0.54 | 1.84 | 19 |
| Chr05 | 337 | 236.42 | 0.70 | 100.32 | 0.30 | 3.36 | 100.37 | 0.30 | 3.36 | 0 |
| Chr06 | 191 | 125.96 | 0.66 | 121.13 | 0.63 | 1.58 | 114.54 | 0.60 | 1.67 | 0 |
| Chr07 | 211 | 144.82 | 0.69 | 93.40 | 0.44 | 2.26 | 92.85 | 0.44 | 2.27 | 15 |
| Chr08 | 282 | 144.06 | 0.51 | 123.50 | 0.44 | 2.28 | 116.02 | 0.41 | 2.43 | 0 |
| Chr09 | 215 | 172.51 | 0.80 | 81.01 | 0.38 | 2.65 | 75.76 | 0.35 | 2.84 | 0 |
| Chr10 | 216 | 138.03 | 0.64 | 111.03 | 0.51 | 1.95 | 108.89 | 0.50 | 1.98 | 0 |
| Chr11 | 279 | 209.69 | 0.75 | 114.02 | 0.41 | 2.45 | 114.85 | 0.41 | 2.43 | 0 |
| Chr12 | 284 | 147.02 | 0.52 | 102.73 | 0.36 | 2.76 | 102.98 | 0.36 | 2.76 | 0 |
| Chr13 | 215 | 148.33 | 0.69 | 103.96 | 0.48 | 2.07 | 108.00 | 0.50 | 1.99 | 0 |
| At | 3049 | 1932.50 | 0.63 | 1335.68 | 0.44 | 2.28 | 1279.46 | 0.42 | 2.38 | 104 |
| Chr14 | 246 | 142.88 | 0.58 | 70.52 | 0.29 | 3.49 | 66.00 | 0.27 | 3.73 | 1 |
| Chr15 | 275 | 127.11 | 0.46 | 64.16 | 0.23 | 4.29 | 59.96 | 0.22 | 4.59 | 6 |
| Chr16 | 231 | 140.51 | 0.61 | 57.62 | 0.25 | 4.01 | 54.92 | 0.24 | 4.21 | 0 |
| Chr17 | 174 | 120.94 | 0.70 | 53.08 | 0.31 | 3.28 | 50.46 | 0.29 | 3.45 | 0 |
| Chr18 | 194 | 111.72 | 0.58 | 64.22 | 0.33 | 3.02 | 58.18 | 0.30 | 3.33 | 0 |
| Chr19 | 329 | 217.86 | 0.66 | 63.89 | 0.19 | 5.15 | 58.92 | 0.18 | 5.58 | 44 |
| Chr20 | 237 | 143.26 | 0.60 | 67.54 | 0.28 | 3.51 | 61.33 | 0.26 | 3.86 | 0 |
| Chr21 | 255 | 207.84 | 0.82 | 72.79 | 0.29 | 3.50 | 64.97 | 0.25 | 3.92 | 7 |
| Chr22 | 201 | 128.96 | 0.64 | 56.4 | 0.28 | 3.56 | 47.19 | 0.23 | 4.26 | 1 |
| Chr23 | 198 | 138.10 | 0.70 | 53.07 | 0.27 | 3.73 | 47.45 | 0.24 | 4.17 | 11 |
| Chr24 | 212 | 152.03 | 0.72 | 69.47 | 0.33 | 3.05 | 63.40 | 0.30 | 3.34 | 25 |
| Chr25 | 194 | 138.72 | 0.72 | 67.61 | 0.35 | 2.87 | 59.55 | 0.31 | 3.26 | 46 |
| Chr26 | 214 | 161.53 | 0.75 | 62.69 | 0.29 | 3.41 | 57.13 | 0.27 | 3.75 | 62 |
| Dt | 2960 | 1931.47 | 0.65 | 823.06 | 0.28 | 3.60 | 749.44 | 0.25 | 3.95 | 203 |
| Total | 6009 | 3863.97 | 0.64 | 2158.74 | 0.36 | 2.78 | 2028.90 | 0.34 | 2.96 | 307 |
|  |  |  |  |  |  |  |  |  |  |  |

| **Supplementary Table 6. Inversions between *G. hirsutum*(AD1) and *G. barbadense*(AD2) genomes.** | | | | | | | | | |
| --- | --- | --- | --- | --- | --- | --- | --- | --- | --- |
|  | ***G. hirsutum*** | | | |  | ***G. barbadense*** | | | |
| **Chr.** | **Physical interval(Mb)** | **Block Length (Mb)** | **Chr. Length (Mb)** | **% of Chr.** |  | **Physical interval(Mb)** | **Block Length (Mb)** | **Chr. Length (Mb)** | **% of Chr.** |
| A01 | 21.86-22.90 | 1.04 | 99.88 | 1.04 |  | 21.26-23.11 | 1.85 | 63.84 | 2.90 |
| A02 | 18.94-20.31 | 1.37 | 83.45 | 1.64 |  | 18.36-19.70 | 1.34 | 98.77 | 1.36 |
| A07 | 28.95-30.51 | 1.56 | 78.25 | 1.99 |  | 30.66-31.98 | 1.32 | 93.49 | 1.41 |
| A09 | 48.27-50.74 | 2.47 | 75.00 | 3.29 |  | 49.01-50.90 | 1.89 | 76.81 | 2.46 |
| A11 | 67.89-71.38 | 3.49 | 93.32 | 3.74 |  | 87.63-89.66 | 2.03 | 115.13 | 1.76 |
| A12 | 19.07-22.24 | 3.17 | 87.48 | 3.62 |  | 24.16-29.77 | 5.61 | 103.35 | 5.43 |
| A13 | 22.76-25.25 | 2.49 | 79.96 | 3.11 |  | 28.87-40.93 | 12.06 | 108.51 | 11.11 |
| A13 | 62.98-64.22 | 1.24 | 79.96 | 1.55 |  | 91.12-92.32 | 1.20 | 108.51 | 1.11 |
| D01 | 21.58-22.86 | 1.28 | 61.46 | 2.08 |  | 24.61-27.17 | 2.56 | 60.1 | 4.26 |
| D02 | 36.02-39.85 | 3.83 | 67.28 | 5.69 |  | 33.62-37.06 | 3.44 | 66.13 | 5.20 |
| D03 | 18.77-22.13 | 3.36 | 46.69 | 7.20 |  | 25.65-27.17 | 1.52 | 50.68 | 3.00 |
| D03 | 30.81-33.99 | 3.18 | 46.69 | 6.81 |  | 36.87-39.52 | 2.65 | 50.68 | 5.23 |
| D07 | 0.38-1.65 | 1.27 | 55.31 | 2.30 |  | 0.25-1.41 | 1.16 | 55.25 | 2.10 |
| D08 | 10.60-12.12 | 1.52 | 65.89 | 2.31 |  | 10.64-12.87 | 2.23 | 63.71 | 3.50 |
| D08 | 32.31-33.92 | 1.61 | 65.89 | 2.44 |  | 24.22-25.69 | 1.47 | 63.71 | 2.31 |
| D08 | 37.77-40.17 | 2.40 | 65.89 | 3.64 |  | 35.82-37.67 | 1.85 | 63.71 | 2.90 |

| **Supplementary Table 7. Genome variations identified via comparative mapping against D-genome pseudomolecules.** | | | | | | | | | | | | |
| --- | --- | --- | --- | --- | --- | --- | --- | --- | --- | --- | --- | --- |
| **Allotetraploid cotton** | | | | |  | ***G. raimondii*** | | | | | | **Events** |
| **Chr.** | **Genetic Interval(cM)** | **Size (cM)** | **Total size (cM)** | **% of Chr** |  | **Chr.** | **Physical interval(Mp)** | **Size (Mb)** | **Total size (Mb)** | **% of Chr** | |  |
| Chr02 | 49.85-54.16 | 4.31 | 106.76 | 4.04 |  | Chr03 | 17.16-28.56 | 11.40 | 45.77 | | 24.91 | Inversion |
| Chr02 | 54.16-55.23 | 1.07 | 106.76 | 1.00 |  | Chr03 | 10.02-13.00 | 2.88 | 45.77 | | 6.29 | Inversion |
| Chr02 | 55.69-62.90 | 7.21 | 106.76 | 6.75 |  | Chr03 | 4.99-8.18 | 3.19 | 45.77 | | 6.98 | Inversion |
| Chr03 | 58.54-63.66 | 5.12 | 132.42 | 3.87 |  | Chr03 | 29.94-32.81 | 2.87 | 45.77 | | 6.28 | Inversion |
| Chr05 | 173.59-177.89 | 4.30 | 236.42 | 1.82 |  | Chr12 | 11.54-16.31 | 4.77 | 35.43 | | 13.46 | Inversion |
| Chr08 | 52.08-66.42 | 14.34 | 144.06 | 9.95 |  | Chr04 | 26.09-37.00 | 4.49 | 62.18 | | 17.54 | Inversion |
| Chr09 | 40.23-45.70 | 5.47 | 172.51 | 3.17 |  | Chr06 | 19.27-25.35 | 6.08 | 51.07 | | 11.90 | Inversion |
| Chr10 | 41.08-49.85 | 8.77 | 138.03 | 6.36 |  | Chr11 | 7.03-9.06 | 2.03 | 62.68 | | 3.25 | Inversion |
| Chr10 | 50.56-59.62 | 9.06 | 138.03 | 6.56 |  | Chr11 | 10.32-14.85 | 4.53 | 62.68 | | 7.22 | Inversion |
| Chr10 | 62.86-80.21 | 17.35 | 138.03 | 12.57 |  | Chr11 | 17.30-43.90 | 26.60 | 62.68 | | 42.44 | Inversion |
| Chr11 | 124.40-130.89 | 6.49 | 209.69 | 3.10 |  | Chr07 | 24.86-35.32 | 10.46 | 60.98 | | 17.15 | Inversion |
| Chr12 | 36.81-40.06 | 3.25 | 147.02 | 2.21 |  | Chr08 | 3.75-5.92 | 2.17 | 57.13 | | 3.81 | Inversion |
| Chr13 | 55.09-59.42 | 4.33 | 148.33 | 2.92 |  | Chr13 | 6.41-11.03 | 4.62 | 58.32 | | 7.92 | Inversion |
| Chr17 | 55.54-58.79 | 3.25 | 120.94 | 2.69 |  | Chr03 | 16.36-27.51 | 11.15 | 45.77 | | 24.35 | Inversion |
| Chr18 | 63.67-67.98 | 4.31 | 111.72 | 3.85 |  | Chr13 | 48.55-50.98 | 2.43 | 58.32 | | 4.16 | Inversion |
| Chr19 | 185.72-196.30 | 10.58 | 217.86 | 4.86 |  | Chr09 | 67.40-68.35 | 0.95 | 70.71 | | 1.34 | Inversion |
| Chr23 | 35.69-38.37 | 2.68 | 138.10 | 1.94 |  | Chr06 | 19.27-24.87 | 5.60 | 51.07 | | 10.97 | Inversion |
| Chr04 | 49.41-50.48 | 1.07 | 96.77 | 1.11 |  | Chr09 | 49.96-64.11 | 14.14 | 70.71 | | 20.00 | Simple translocation |
| Chr22 | 59.54-76.17 | 16.63 | 128.96 | 12.89 |  | Chr09 | 53.88-66.12 | 12.24 | 70.71 | | 17.31 | Simple translocation |
| Chr11 | 130.89-133.04 | 2.15 | 209.69 | 1.03 |  | Chr03 | 13.17-14.86 | 1.69 | 60.98 | | 2.78 | Simple translocation |
| Chr21 | 126.61 | - | 207.84 | - |  | Chr03 | 13.58-15.76 | 2.18 | 45.77 | | 4.76 | Simple translocation |
| Chr13 | 60.49-63.74 | 3.25 | 148.33 | 2.19 |  | Chr01 | 48.77-51.73 | 2.96 | 55.87 | | 5.30 | Simple translocation |
| Chr18 | 45.23 | - | 111.72 | - |  | Chr01 | 48.67-51.73 | 3.06 | 55.87 | | 5.48 | Simple translocation |
| Chr17 | 55.54 | - | 120.94 | - |  | Chr02 | 5.01-6.93 | 1.92 | 58.32 | | 3.29 | Simple translocation |
| Chr02 | 0-49.85 | 49.85 | 106.76 | 46.69 |  | Chr05 | 0.18-27.78 | 27.6 | 64.14 | | 43.03 | Reciprocal translocation |
| Chr03 | 0-63.66 | 63.66 | 132.42 | 48.07 |  | Chr03 | 29.94-45.39 | 15.45 | 45.77 | | 33.75 | Reciprocal translocation |
| Chr04 | 0-49.41 | 49.41 | 96.77 | 51.06 |  | Chr09 | 45.96-70.42 | 24.45 | 70.71 | | 34.58 | Reciprocal translocation |
| Chr05 | 173.59-236.42 | 62.83 | 236.42 | 26.57 |  | Chr12 | 0.05-16.31 | 16.26 | 35.43 | | 45.90 | Reciprocal translocation |
|  |  |  |  |  |  |  |  |  |  | |  |  |

| **Supplementary Table 8. Breaking manner of two reciprocal translocations.** | | | | | | | | | | | | | | |
| --- | --- | --- | --- | --- | --- | --- | --- | --- | --- | --- | --- | --- | --- | --- |
|  |  |  |  |  |  |  |  |  |  |  |  |  |  |  |
| **Genetic map** | | |  | ***G. hirsutum*** | | |  | ***G. barbadense*** | | |  | ***G. raimondii*** | | |
| **Chr.** | **Position** | **Coverage** |  | **Chr.** | **Position** | **Coverage** |  | **Chr.** | **Position** | **Coverage** |  | **Chr.** | **Position** | **Coverage** |
|  | **(cM)** | **(%)** |  |  | **(Mb)** | **(%)** |  |  | **(Mb)** | **(%)** |  |  | **(Mb)** | **(%)** |
| Chr02 | 0-49.85 | 46.69 |  | A02 | 0.05-34.72 | 41.55 |  | A02 | 0-34.28 | 34.71 |  | Chr05 | 0.08-31.31 | 31.68 |
| Chr02 | 49.85-106.76 | 53.31 |  | A02 | 38.12-83.26 | 54.09 |  | A02 | 35.70-98.72 | 63.80 |  | Chr03 | 0.11-27.85 | 60.61 |
| Chr03 | 0-63.65 | 47.84 |  | A03 | 0.14-21.28 | 21.09 |  | A03 | 0.47-23.79 | 22.38 |  | Chr03 | 29.94-45.39 | 33.75 |
| Chr03 | 63.65-132.42 | 52.16 |  | A03 | 24.73-100.24 | 75.31 |  | A03 | 28.85-103.86 | 71.99 |  | Chr05 | 17.16-64.08 | 59.07 |
| Chr04 | 0-50.48 | 52.16 |  | A04 | 0.24-42.05 | 66.46 |  | A04 | 0.47-61.28 | 75.66 |  | Chr09 | 48.15-70.42 | 31.49 |
| Chr04 | 50.48-96.77 | 47.84 |  | A04 | 44.60-62.79 | 28.91 |  | A04 | 62.15-80.17 | 22.42 |  | Chr12 | 23.12-35.35 | 34.52 |
| Chr05 | 0-173.592 | 73.43 |  | A05 | 0.17-65.52 | 64.88 |  | A05 | 0.09-66.35 | 65.78 |  | Chr09 | 0.14-45.16 | 63.67 |
| Chr05 | 174.67-236.42 | 26.12 |  | A05 | 67.38-91.59 | 24.03 |  | A05 | 68.70-100.46 | 31.53 |  | Chr12 | 0.05-16.31 | 45.89 |

| **Supplementary Table 9. Distribution of the overlapping region of A02 and A03 compared with Gr_Chr05.** | | | | | | | | |
| --- | --- | --- | --- | --- | --- | --- | --- | --- |
| ***G. raimondii*** | | | | |  | ***G. hirsutum*** | | |
| **Chr.** | **Interval (bp)** | **Block** | **Region.** | **%** |  | **Chr.** | **Interval (bp)** | **Length** |
|  |  | **Length(Mb)** | **Length(Mb)** |  |  |  |  | **(Mb)** |
| Gr_Chr05 | 16774923-20312487 | 3.54 | 14.53 | 24.36 |  | Gh_A03 | 22562694-28166371 | 5.61 |
| Gr_Chr05 | 20972859-23556739 | 2.58 |  | 17.76 |  | Gh_A02 | 23016284-29190843 | 6.17 |
| Gr_Chr05 | 24312256-25427142 | 1.11 |  | 7.64 |  | Gh_A03 | 29303470-31143939 | 1.84 |
| Gr_Chr05 | 25802494-26359746 | 0.56 |  | 3.85 |  | Gh_A02 | 32787168-33933707 | 1.15 |
| Gr_Chr05 | 26854591-27145989 | 0.29 |  | 2.00 |  | Gh_A03 | 33473427-35320468 | 1.85 |
| Gr_Chr05 | 28067397-28477442 | 0.41 |  | 2.82 |  | Gh_A02 | 35193448-36201948 | 1.01 |
| Gr_Chr05 | 30829290-31010849 | 0.18 |  | 1.24 |  | Gh_A03 | 31385496-34298987 | 2.91 |
| Gr_Chr05 | 31306757-31309785 | 0.003 |  | 0.02 |  | Gh_A02 | 32210967-32213545 | 0.003 |
